# Artificial light at night reverses monthly foraging pattern under simulated moonlight

**DOI:** 10.1098/rsbl.2022.0110

**Published:** 2022-07-27

**Authors:** Svenja Tidau, Jack Whittle, Stuart R. Jenkins, Thomas W. Davies

**Affiliations:** ^1^ School of Biological and Marine Sciences, University of Plymouth, Plymouth PL4 8AA, UK; ^2^ School of Ocean Sciences, University of Bangor, Menai Bridge LL59 5AB, UK

**Keywords:** artificial light at night, foraging, lunar biology, lunar cycles, moonlight, sensory ecology

## Abstract

Mounting evidence shows that artificial light at night (ALAN) alters biological processes across levels of organization, from cells to communities. Yet, the combined impacts of ALAN and natural sources of night-time illumination remain little explored. This is in part due the lack of accurate simulations of the complex changes moonlight intensity, timing and spectra throughout a single night and lunar cycles in laboratory experiments. We custom-built a novel system to simulate natural patterns of moonlight to test how different ALAN intensities affect predator–prey relationships over the full lunar cycle. Exposure to high intensity ALAN (10 and 50 lx) reversed the natural lunar-guided foraging pattern by the gastropod mesopredator *Nucella lapillus* on its prey *Semibalanus balanoides*. Foraging decreased during brighter moonlight in naturally lit conditions. When exposed to high intensity ALAN, foraging increased with brighter moonlight. Low intensity ALAN (0.1 and 0.5 lx) had no impact on foraging. Our results show that ALAN alters the foraging pattern guided by changes in moonlight brightness. ALAN impacts on ecosystems can depend on lunar light cycles. Accurate simulations of night-time light cycle will warrant more realistic insights into ALAN impacts and also facilitate advances in fundamental night-time ecology and chronobiology.

## 1. Introduction

Ecological light pollution is now an established field of global change research [1]. Satellite night-time imagery illustrates unequivocally the vast global extent of artificial light at night (ALAN). At least 80% of the world's population is exposed to ALAN [2] and its influence is expanding both in area (2.2% per year) and intensity (1.8% per year) [3]. Mounting evidence shows that ALAN alters biological processes across levels of organization, from cells to communities, and across a range of biomes, taxa and spatial scales [4,5]. Accurate prediction and mitigation of ALAN impacts demand a deeper understanding of how they are modified by other factors that shape the natural night-time light environment and biological adaptations to them.

The moon is the single most important source of environmental night-time illumination. It drives large-scale ecosystem processes and a diverse array of physiological and behavioural rhythms [6], the most widely known being lunar entrained global synchronized mass spawning in corals [7]. Lunar-driven phenological life-history events such as reproduction and migration are found across the animal kingdom in marine [8–11], terrestrial [12,13] and freshwater [14,15] habitats across the globe. Lunar rhythms influence organisms' growth [16] and activity patterns [12]. Moonlight intensity affects communication [13], orientation [17] and risk–reward trade-offs [18,19]. Recent research suggests that ALAN interferes with lunar guided migration [20], orientation [21], sleep time [22] and reproduction [23] at intensities similar to natural moonlight.

**Figure 1.**
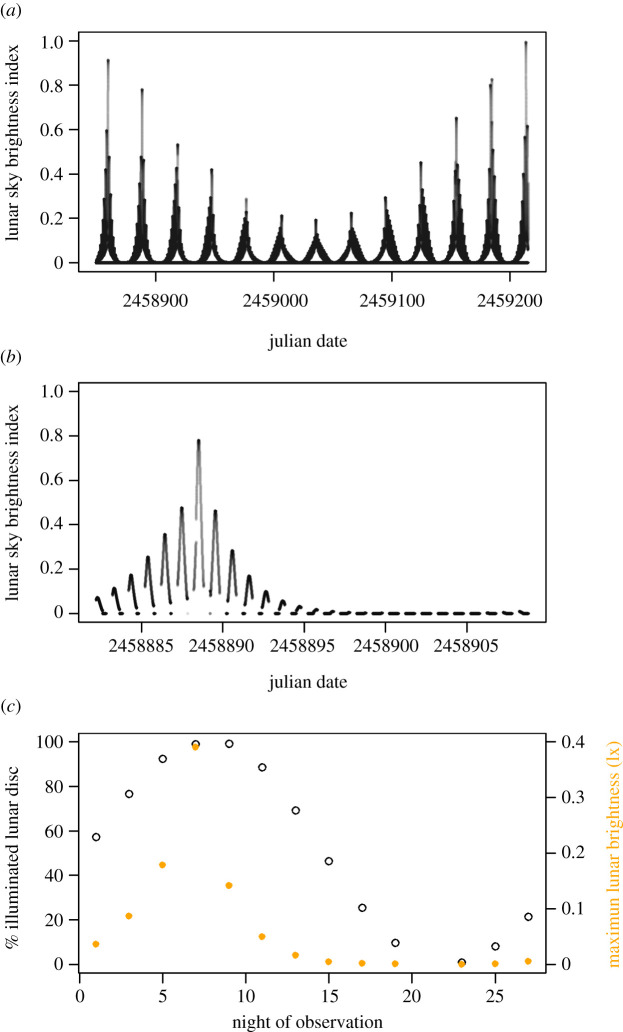
Lunar cycle in nature and in the laboratory for Menai Bridge, UK. (*a*) Lunar cycle in nature over 12 month in 2020 (astronomical unit Julian date, days elapsed since 1 January 4713 BC) as lunar sky brightness index (normalized to 1 = 0.5 lx). (*b*) Lunar cycle in nature over the course of the experiment (2 February–2 March) as lunar sky brightness index (normalized to 1 = 0.5 lx). (*c*) Lunar cycle in the laboratory as percentage illuminated disc following a sinosodial pattern (circles, left *y*-axis) and as maximum lunar brightness (in lx) (closed points, right *y*-axis).

Current evidence of ALAN disrupted lunar biology is often limited to characterizing only the moon phases, which do not reflect the lunar cues organisms are likely to detect in the wild. Moon phases describe the lunar cycle as the portion of illuminated lunar disc as observed from the Earth and suggest a sinusoidal pattern in lunar intensity when, in reality, the pattern of changes in lunar brightness throughout a cycle follows extreme peaks and troughs as the moon transits the sky (figure 1) [24]. Lunar intensity varies throughout the night, with day, month, year and enneadecaeteris (the approx. 19 year metonic cycle) for any location and time, owing to variations in lunar phase angle, altitude and atmospheric scattering (figure 1*a*,*b*) [25,26].

We built a novel system that allows us to quantify the ecological impacts of ALAN over a full lunar cycle simulating the timing and intensity of moonlight as experienced in nature. Rather than simulating sinusoidal changes in lunar phases, our system simulates natural night-time conditions for a specified location and date (figure 1*b*,*c*). We exposed the gastropod mesopredator *Nucella lapillus* to a range of seven ALAN intensities, from low levels within the range of natural lunar light intensities (0.1 and 0.5 lx), up to levels (10 and 50 lx) similar to those experienced by organisms close to ports, harbours and street lights [27]. We measured the foraging probability of *Nucella* on its prey, the barnacle *Semibalanus balanoides*, over an entire lunar cycle. We asked (a) whether ALAN impacts vary over the lunar cycle; (b) whether ALAN impacts are expressed during the night- or daytime; and (c) whether ALAN impacts change over the duration of the experiment (due to acclimation).

## 2. Methods

*Nucella* were collected from an artificial light naive shore on the island of Anglesey, UK (53°11'6″ N, 4°29'35″ W) on 31 January 2020, and transported to the School of Ocean Sciences, Menai Bridge, UK (53°13'57″ N, 4°10'22″ W). Individuals were sexed, and male *Nucella* marked for individual identification and assigned to one of seven light treatment chambers (no ALAN, 0.1 lx, 0.5 lx, 1 lx, 10 lx, 10 lx mitigation and 50 lx). Each 0.16 m^3^ light treatment chamber contained three 2 l clear Perspex tanks (*n* = 21; 20 L ×12 W × 13 H cm), each of which housed four individuals (*n* = 84) in 0.5 µm filtered UV-irradiated seawater changed every second day. *Nucella* had ad libitum access to rocks (*ca* 50 cm^2^ surface area, less than 5% of the total 1072 cm^2^ available tank surface area) covered with their prey, the barnacle *Semibalanus balanoides*, replaced every 4 days.

The natural daylight and moonlight regimes experienced by *Nucella* on their native shore were simulated in each chamber. Daylight was simulated using an Aquaray Natural Daylight Tile set at 5000 lx (mean 4781 lx ±5%) and the BioLumen Control Unit (Tropical Marine Centre, UK) programmed in real time to the sunrise and sunset times of Menai Bridge, UK (53°13'57″ N, 4°10'22″ W). To evenly diffuse the light and minimize bright spots [4], the daylight tile was covered by 3 mm frosted Perspex. Moonlight regimes were simulated using a bank of 2700–3500 K 1.2 cd LEDs housed within diffusing spheres to minimize light spots. Natural moonlight regimes were simulated using a pulse width modulated signal (scale 0–100%) applied to the 5 V output of Raspberry Pi 3 model B+, with maximum lunar brightness set to 0.5 lx (observable within 2020, figure 1*a*). Lunar brightness was adjusted from a look up table (1 min resolution) of Zenith Sky Brightness modelled for Menai Bridge. Modelling followed [27] whereby the moon's sky position and phase angle are calculated from the time, date and geocentric coordinates of location (CRAN: astrolib). The Zenith Sky Brightness is then modelled accounting for lunar phase, altitude, opposition, parallax and atmospheric scattering according to Krisciunas & Schaefer [28]. Uniquely, in comparison to previous lunar simulations under experimental laboratory settings, our system captures variability in night-time lighting as the moon transits the sky [25,26]. The spectrum of moonlight changes throughout the night with lunar phase and elevation [29,30]. As with twilight, this persists to be technically challenging [4] and hence was not manipulated. ALAN was simulated between dawn and dusk (triggered using a CellOptick 12 V photocell) using Aquaray cool white FlexiLED strips (Tropical Marine Centre, UK), with brightness controlled using voltage dimming. As the lens eyes [31] of aquatic gastropods typically show peak spectral sensitivity from 470 to 505 nm [32,33], we evaluated a potential mitigation solution using a long bandpass (510–2200 nm) yellow acrylic filter (www.knightoptical.com), which minimizes blue wavelengths prominent in LEDs. This was implemented in one of two 10 lx treatment chambers.

Behaviour was observed over one lunar cycle between 2 February and 2 March 2020 using infrared time-lapse photography. GoPro Hero 4 cameras fitted with infrared pass lenses were programmed with Blink Time Lapse Controllers for GoPros (CamDo, USA) to take one photo every 5 min for 24 h every second night over the 28 day period (= 289 photos per 24 h, for each of the seven treatments, each of the 12 individually marked animals per treatment, for 13 nights of observation = up to 315 588 photos. Owing to a charging error, there are no observations for day 21, see figure 1*c*). This sampling frequency allowed the recording system to last for 24 h (from 15.00 day 1 to 15.00 day 2) and to capture 13 nights without interruption over one lunar cycle. Images were down-sampled from colour to 8-bit greyscale with ImageJ. Brightness and contrast were adjusted to maximize visibility. Images were converted into a single time-lapse video for each 24 h. Owing to naturally high levels of inactivity in *Nucella*, we classed their behaviour as either foraging (when sitting on the rock with barnacles) or not foraging (when not sitting on a rock). This is a common metric for gastropod foraging and avoids disturbing animals [34–37]. We also recorded whether the behaviour occurred day and/or night-time leading to two data points per individual per video. Owing to the persistent technical challenges in simulating twilight timing, spectra and intensity [4], we excluded footage taken over dusk and dawn.

We quantified whether *Nucella*'s foraging activity (binary: Foraging/Not Foraging) was affected by ALAN (categorical: 0, 0.1, 0.5, 1, 10, 10 mitigation, 50 lx) in interaction with either (a) moonlight intensity (continuous: maximum lunar brightness per night; figure 1*c*), (b) time of day (categorical: night or day), and (c) experimental day, i.e. night of observation (continuous: night 1–27) using R (version 4.1.2). The latter explored potential collinear effects that may arise due to *Nucella* acclimatizing. To find the most parsimonious model, we first fitted a global binomial generalized linear model (GLM) with the following interactions: ALAN*Moonlight + ALAN*NightofObservation + ALAN*TimeofDay. Next, we used the dredge function (CRAN: MuMIn) which automates model selection through subsetting the maximum model based on model weights derived from Akaike's information criterion (AIC). The model explaining less than 99% of the response based on weight and the lowest AICs included ALAN*Moonlight + ALAN*NightofObservation + TimeofDay (see electronic supplementary material, table S1 for all models). This most parsimonious GLM was compared to an intercept only model for validation using a likelihood ratio test [38]. Since the GLM explained significantly more variance in the response than the intercept only model (*χ^2^* = −80.58, d.f. = 21, *p* < 0.001), the GLM was refitted as generalized linear mixed effects (GLMM) model (CRAN: lme4). Snail ID was nested in tank as a random factor to account for the experimental design. The significance of the GLMM parameters was quantified using the Type III ANOVA approach of stepwise model selection [39]. Again, models were compared using likelihood ratio tests. Significant difference between treatment levels were quantified by pairwise comparisons using the emtrends function (CRAN: emmeans) which allows the inclusion of a numerical predictor (here Moonlight) interacting with a factorial predictor (here ALAN). We did not adjust the *p*-value to avoid inflating the Type I error. The predicted relationships and their 95% intervals were modelled for visual presentation using the predictInterval function (CRAN: merTools).

## 3. Results

**Figure 2.**
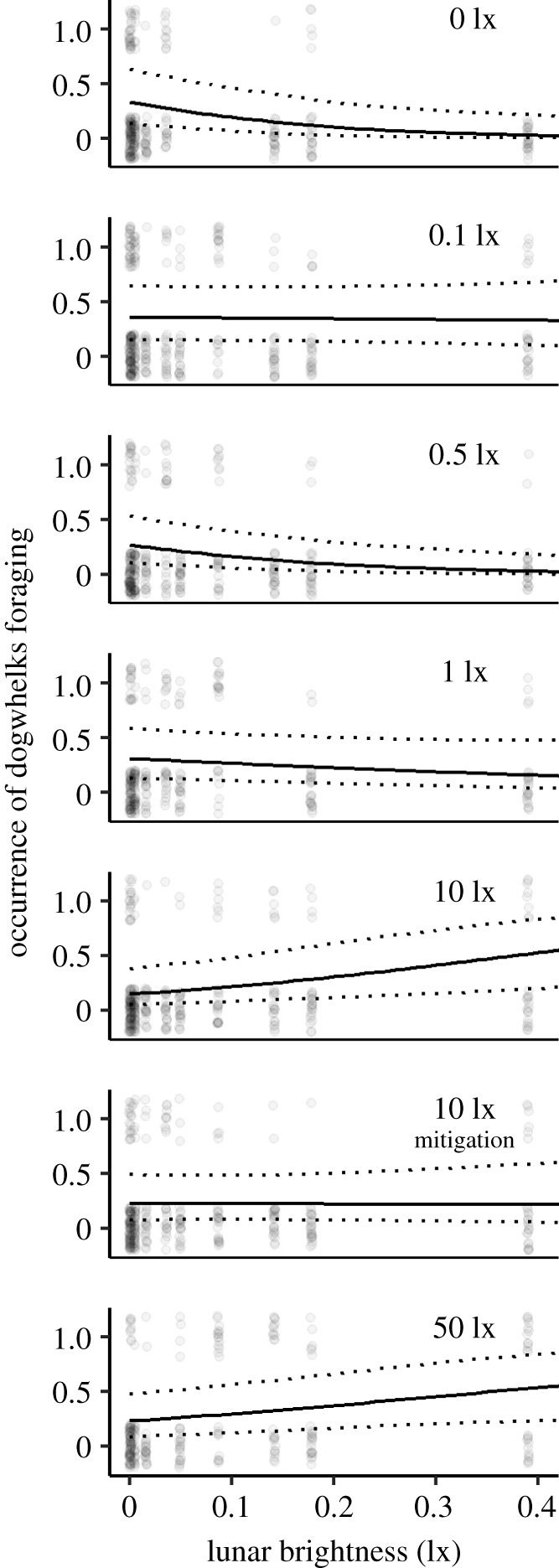
The interactive impact of different ALAN intensities and maximum lunar brightness on foraging occurrence in *Nucella lapillus*. The figure shows the raw data (jittered dots), predicted relationships (solid line) and 95% prediction intervals (dotted lines).

Foraging activity was influenced by the nightly maximum lunar brightness, however, the direction of this relationship was significantly affected by exposure to different ALAN treatment levels (ALAN*Moonlight: *χ^2^* = 33.67, d.f. = 6, *p* < 0.001; figure 2 and table 1; electronic supplementary material, table S2). Under natural night-time light simulations (no ALAN), *Nucella* were less likely to forage on brightly moonlit nights, while under high intensity ALAN (10 and 50 lx), *Nucella* were more likely to forage on brightly moonlit nights (figure 2). Foraging activity in *Nucella* exposed to the mitigation treatment (filtered out light under 510 nm) differed from both the 10 lx treatment and control conditions (figure 2; electronic supplementary material, table S2).The impact of ALAN on foraging was affected by night of observation (*χ^2^* = 17.29, d.f. = 6, *p* = 0.008; table 1; electronic supplementary material, table S3). Time of day had no effect on foraging (*χ^2^* = 0.18, d.f. = 1, *p* = 0.670).

**Table 1.** The impact of ALAN, lunar brightness, night of observation and time of day on foraging in *Nucella lapillus*. Significant main effects and interactions are in italics.

main effects and interactions	*χ^2^*	d.f.	*p*
ALAN*Moonlight	*33.67*	*6*	*< 0.001*
ALAN*Night of Observation	*17.29*	*6*	*0.008*
ALAN	4.89	6	0.558
Moonlight	1.74	1	0187
*Night of Observation*	*10.41*	*1*	*0.001*
Time of Day	0.18	1	0.670

## 4. Discussion

ALAN is known to affect lunar-guided reproductive phenology [23], orientation [20,21] and community structure [19]. Our results demonstrate that ALAN can also alter temporal patterns in foraging tuned to the naturally changing nightly lunar brightness throughout the lunar cycle.

Under the simulation of natural moonlight, *Nucella* foraged less with increasing moonlight intensity. Suppressed activity on brighter moonlit nights is a common pattern driven by risk–reward trade-offs [12,18,19]. High-intensity ALAN levels, however, reversed this pattern. *Nucella* was more likely to forage during brighter moonlit nights under ALAN intensities of 10 and 50 lx. The gastropod sensory system allows *Nucella* to use chemical and visual channels to detect prey (mussels and barnacles) and predators (crabs and birds) [33,40]. The high-intensity ALAN treatments masked the maximum lunar brightness attained on any night during the experiment (0.39 lx) and could have been bright enough for *Nucella* to visually exclude predation risk. This interpretation aligns with previous observations of *Nucella* foraging in the presence of predator olfactory cues when exposed to ALAN but not in dark control treatments [41]. ALAN intensities of 0.5 and 1 lx, which are similar to artificial skyglow [20], could be too dark to allow accurate visual assessment of the environment and risk perception.

A growing body of evidence indicates that ALAN has notable impacts on lunar-guided biological processes [18–21,23]. Here we show that ALAN impacts also depend on natural regimes of lunar brightness. The brightness of naturally lit nights is a function of lunar phase, altitude and scattering, yet studies investigating both ALAN impacts and chronobiological responses to moonlight simulate the sinusoidal pattern of lunar phase at best [26,42,43]. Moonlight intensity does not change in a sinusoidal pattern, as suggested by lunar phases, which give the portion of illuminated lunar disc as observed from the Earth. The full moon is 1.3 times brighter than can be accounted for solely by the increase in percentage of illuminated lunar disc due to the so-called lunar opposition effect. This phenomenon describes the nonlinear intensity increase with decreasing phase angle [25,27,28]. In nature, animals hardly experience maximum lunar brightness between 0.2 and 0.4 lx. To quantify biologically relevant ALAN impacts on organisms over a lunar cycle requires simulating the lunar intensity accurately. Resolving technical challenges in mimicking the spectral composition of moonlight [28,30] will facilitate further mechanistic insight also into crepuscular processes [6,7] and ALAN disruptions to them. ALAN research is increasingly embedded into a multisensory pollution approach [19,44] to assess its interactions with other anthropogenic stressors like noise [45,46] and warming [47]. Future research that aims to facilitate a better understanding of anthropogenic impacts on wildlife should also consider how these interact with natural factors. For ALAN research, this means first and foremost lunar cycles described by temporal variability in moonlight intensity through the night, month, year and enneadecaeteris. Our results highlight the importance of accounting for moonlight when investigating ALAN impacts. In the laboratory setting, this means accurately simulating moonlight. Doing so will provide novel mechanistic insights in the fields of ecological light pollution, visual ecology, night-time ecology and chronobiology, and improve the application of experimental results to the real world.

## Data Availability

The data of this study, READme file and R code are available as electronic supplementary material [48].
